# Investigation of Early Stage of Carbon Nanotube Growth on Plasma-Pretreated Inconel Plates and Comparison with Other Superalloys as Substrates

**DOI:** 10.3390/nano10081595

**Published:** 2020-08-14

**Authors:** Eui-Chul Shin, Byeong-Joo Lee, Sung-Il Jo, Goo-Hwan Jeong

**Affiliations:** Department of Advanced Materials Science and Engineering, Kangwon National University, Chuncheon, Gangwon-do 24341, Korea; shinec@jnltech.co.kr (E.-C.S.); leebj@kangwon.ac.kr (B.-J.L.); sungil107@kangwon.ac.kr (S.-I.J.)

**Keywords:** carbon nanotubes, plasma treatment, superalloys, invar 42, Hastelloy C276, Inconel 600

## Abstract

We investigate the early stage of carbon nanotube (CNTs) growth on Inconel 600 to address the effect of pretreatments such as annealing and plasma pretreatment on growth behavior. In addition, we compare the growth results to other Ni-based superalloys including Invar 42 and Hastelloy C276. The growth substrates were prepared using mechanical polish, thermal annealing and plasma pretreatment. The air annealing was performed at 725 °C for 10 min and plasma pretreatment was subsequently undergone with 10.5 W at 500 °C for 30 min. The annealed and plasma-pretreated substrates exhibited different surface morphologies on the surface and enhanced growth behavior of CNT was observed from the region of particulate surface. The optimized growth temperature, which produces the highest CNT height, was determined at 525 °C for Ni and Inconel 600 and 625 °C for Invar 42 and Hastelloy C276 substrates. The difference of optimal growth temperature is expected to the existence of high temperature elements such as Mn or Mo in the alloys. X-ray diffraction spectroscopy revealed that the formation of roughened oxide layers caused by the pretreatments would promote the nucleation and growth of the CNTs.

## 1. Introduction

Carbon nanotubes (CNT) are attractive materials since they have extraordinary physical, chemical and electrical properties [1]. In order to realize industrial applications, the formation of predesigned CNT structures on specific substrates has also been considered as a crucial requirement. For instance, the growth of CNTs on metallic substrates is highly desirable to apply to some applications such as device interconnections, electrodes, heat dissipation and field emission devices, where superior electric or thermal conductivity is required at the interface between CNT and substrates [2,3,4,5,6,7,8,9,10].

Among the various growth techniques, a chemical vapor deposition (CVD) has been widely employed to grow CNTs because of its relatively easy accessibility and simple operation. It has been known the vapor-liquid-solid mechanism can be applicable as a growth mechanism of CNTs in a CVD. Thus, catalytic metal particles are necessary to dissolve atomic carbon from a feedstock and precipitate as a solid form of carbon to make hexagonal structure of carbon networks [11]. To produce CNTs with specified tube diameter and length, the thickness control of catalytic films is a key factor because the films yields catalytic nanoparticles on the substrate through the de-wetting process during the high temperature CVD process [12,13]. In addition, to grow vertically aligned CNT with quantity, it is essential to deposit a catalytic metal thin films on insulating substrates, such as Al_2_O_3_ or SiO_2_. Here, the separation of catalysts and substrates by the buffer layer prevents the formation of alloys or compounds which are harmful for CNT growth [14,15]. On the other hand, it is very difficult to preserve outstanding conductivity at the interface due to the insulating buffer layers. Thus, the thickness of the buffer layers must be controlled from the viewpoint of directional conductivity from CNTs to substrates which are used as, especially, charge paths in some applications. From the reasons, several works including ours have tried to grow CNTs on metallic substrates to overcome the above-mentioned limitations. For instance, Talapatra et al. reported the CNT growth on Inconel 600 at 770 °C using a ferrocene–xylene CVD method [2]. Pal et al. and Bult et al. also successively reported the follow-up investigation for direct growth of CNT on Inconel 600 substrates [3]. We also reported the highly efficient CNT growth on plasma-pretreated stainless steel substrates and Inconel 600 as well [4,5]. Cu [6] and Ta [7] have also been demonstrated as growth substrates with high electrical conductivity. Again, the main purpose of all the works has been focused on electrical conductivity enhancement between CNTs and substrates for current collector application. In spite of the above-mentioned efforts, further investigations, especially in early stage of CNT growth, are still required to understand the growth behaviors on various metallic substrates which accelerate the realization of industrial applications.

Herein, in order to investigate the effect of substrate pretreatments on CNT growth, the early stage of CNT growth on Inconel 600 surface was scrutinized. We also compare CNT growth results to other Ni-based superalloys, such as Invar 42 and Hastelloy C276. From our previous works, the optimized condition of pretreatments consisting of thermal annealing at 725 °C and plasma pretreatment with Ar gas of 10.5 W at 500 °C was employed and detailed analyses of the substrate surfaces was conducted. After the pretreatments, particulate morphology with relatively high surface roughness was formed over the substrate surface yielding increase of CNT amount. In case of Inconel 600 and Ni sheets, CNT growth was significantly increased compared to that of Invar 42 and Hastelloy C276 substrates. Finally, it is found that the optimized pretreatment involves the formation of roughened oxide surface, which would promote the nucleation and growth of the CNTs.

## 2. Materials and Methods

### 2.1. Substrate Pretreatments and CNT Growth on Ni-Based Superalloys

As for growth substrate of CNTs, we used Invar 42, Hastelloy C276 and Inconel 600 sheets since they commonly contain catalytic metal element, Ni, for CNT growth (Table 1). Thus, we do not need to deposit specific catalytic and oxide buffer layers. A Ni sheet was also used for a comparison. 

In order to investigate the effect of pretreatments on CNT growth, we mechanically polished the purchased sheets using sandpaper to obtain a mirror plane. Thermal annealing was performed using a horizontal quartz-tube furnace (1-inch diameter). Annealing condition was optimized at 725 °C for 10 min under ambient atmosphere. Then, the substrates were successively plasma-treated using a direct current power source with a diode-type parallel electrodes configuration. The Ar (99.999%) was used for plasma ignition and the plasma treatment was performed at 0.5 Torr. The introduced power for the treatment was varied 7.5 W to 14 W and maintained for 30 min at 500 °C.

After the pretreatments, the samples were inserted into a horizontal quartz-tube furnace and the furnace was heated to growth temperatures and held for 10 min. The mixture of Ar (900 sccm) and H_2_ (100 sccm) was introduced during the heating. The growth of CNTs was initiated by the introduction of acetylene (50 sccm), Ar (500 sccm) and H_2_ (500 sccm) gases. The growth temperature was varied from 375 to 725 °C to confirm the optimal growth temperature in each substrate. After the growth for 30 min, the chamber was furnace-cooled to ambient temperature.

### 2.2. Characterization

For characterization of surface morphology and CNT structures, atomic force microscopy (AFM Park Systems X-70 city, Suwon, Korea) in tapping mode operation and scanning electron microscope (SEM, Coxem CX-100, Daejeon, Korea) were used. To directly confirm the CNT structure and chemical elements of catalysts, a transmission electron microscopy (TEM, JEM2100F, Tokyo, Japan) and energy dispersive spectroscopy (EDS, Horiba EX250, Tokyo, Japan) were used. As for TEM sample preparation, as-grown CNTs were dispersed in ethanol using ultrasonication, then, the solution was dropped into a TEM grid and allowed it to dry. Raman spectroscopy (Horiba Aramis, Piscataway, NJ, USA), a versatile and convenient tool for estimating the structural completeness of CNTs by comparing the peak intensities of a structural-disorder-induced peak (D-band, I_D_) around 1350 cm^−1^ and tangential stretching vibration mode of graphite (G-band, I_G_) around 1590 cm^−1^, was also employed [16]. For the Raman analysis, the excitation wavelength of laser was 532 nm and spot size was 1 μm. An X-ray diffractometer (PANalytical X’Pert Pro, Eindhoven, The Netherlands) was employed to address the evolution of constituent of surface layers from as-received to after CNT growth. The analysis was performed with a radiation of Cu-Kα (λ = 1.5418 Å) with a step size of 0.1° and scan speed of 0.005 °/s at room temperature.

## 3. Results

### 3.1. Investigation of Early Stage of CNT Growth on Inconel 600 Surface

**Figure 1:**
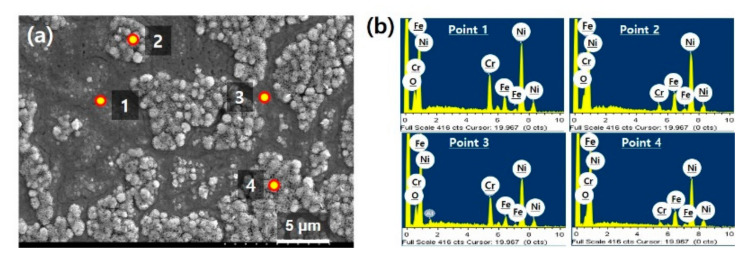
(**a**) SEM image of Inconel 600 substrate surface after air-annealing and plasma pretreatment; (**b**) results of EDS point analysis in (**a**).

In order to address the effect of pretreatments on physical or chemical change of the substrate surface, we first performed a detailed investigation of Inconel surface after the pretreatment. The condition of thermal annealing and plasma treatment was employed from our previous works as mentioned in Section 2.1. Figure 1a is a SEM image of Inconel 600 surface taken after the pretreatment. It was found the substrate was divided into two regions having different morphologies on the surface. While the regions designated points 1 and 3 look flattened, points 2 and 4 indicate roughened regions on the substrate. In addition to the morphologic difference, to identify the difference in chemical composition of two regions, we further performed EDS analyses. The spectra and quantitative result are presented in Figure 1b and Table 2, respectively. It was found the flattened regions (points 1 and 3) had similar chemical composition to that of Inconel 600. However, it was very interesting that the roughened regions (points 2 and 4) show increased Ni and Fe content and significantly reduced Cr content compared to the Inconel and flattened regions. It could be expected that not only the physical change in morphology, but also the chemical change such as compositional segregation occurred during the pretreatments.

**Figure 2:**
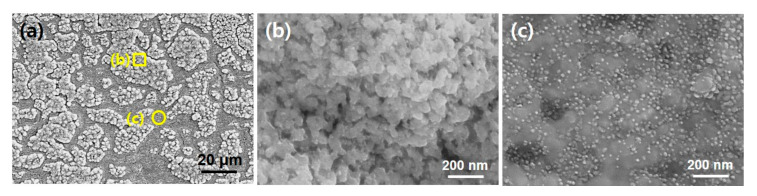
(**a**) Large area SEM view of Inconel 600 substrate surface after air-annealing and plasma pretreatment; (**b**) and (**c**) are high magnification SEM images showing morphologic difference between them.

Next, to explore the morphologic difference in a nanoscale between two regions, we adopted high resolution SEM observation and the results are shown in Figure 2. Figure 2a is a large area SEM image and very similar to Figure 1a. High magnification SEM observation was performed as indicated with yellow square and circle in Figure 2a. Figure 2b,c is highly magnified views of rough and flat regions, respectively. It is interesting the morphologic difference was evident between two regions. In detail, while the aggregated surface morphology was observed in roughened region (Figure 2b), the nanoparticles with 10–20 nm in diameter were uniformly dispersed over the flattened region (Figure 2c).

**Figure 3:**
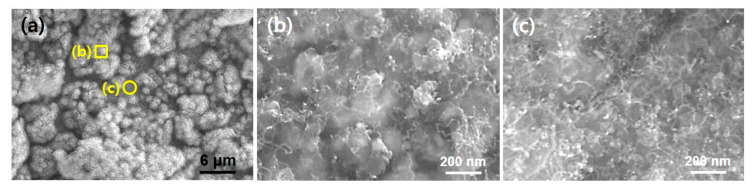
(**a**) Large area SEM view of Inconel 600 substrate surface after 5 s growth of CNT; (**b**) and (**c**) are high magnification SEM images showing different amount of CNT grown.

In general, it has been known the surface morphology is closely related with the final structures of CNT and growth yield as well. Thus, we investigated the behavior of early stage of CNT growth with respect to the different morphology of the pretreated substrate surface as shown in Figure 2. We introduced the feedstock gas for 5 s and instantly evacuated the chamber. Figure 3a shows a large area SEM image of Inconel 600 substrate after the CNT growth for 5 s. As shown in Figure 1a and Figure 2a, the substrate showed both particle like morphology (indicated by yellow square (b)) and flattened surface (indicated by yellow circle (c)). After the 5 s growth, it is found that while the crumbled and very short tubular structures are formed in the roughened region, more dense tubular structures are produced at the flat surface region as shown in Figure 3b,c, respectively. From the SEM images, the tube length appeared below 1 μm and diameter ranges of 10–20 nm which is very similar to that of nanoparticles in Figure 2c. It can be supposed that the tubular structures in Figure 3 would be CNTs grown from the catalytic nanoparticles in Figure 2c.

**Figure 4:**
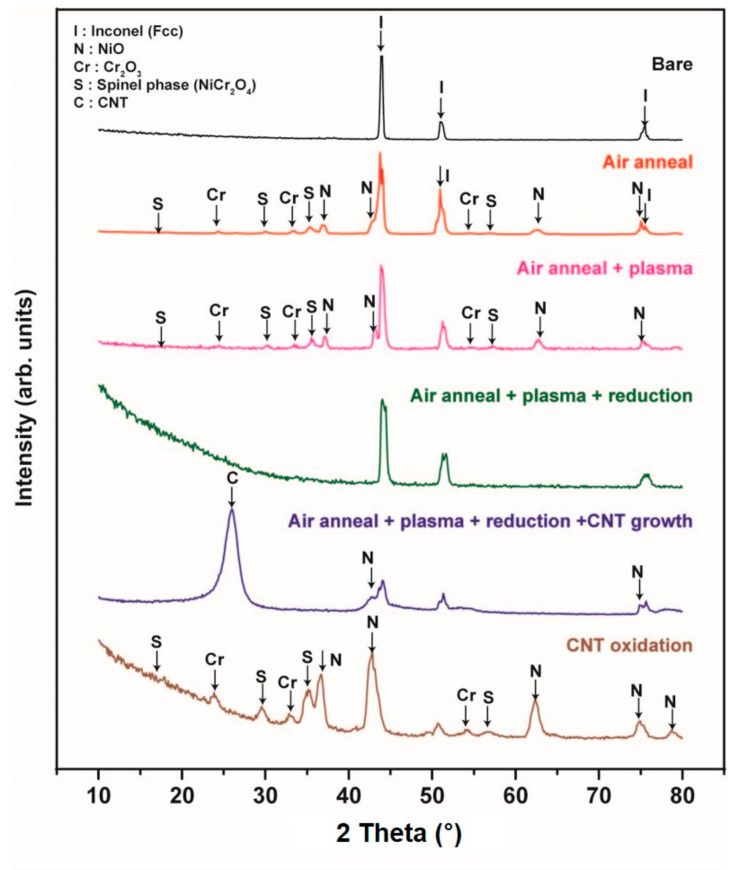
XRD profiles recorded at each step: as-purchased, air annealing, plasma treatment, reduction before growth, CNT growth and CNT oxidation after growth.

Figure 4 shows a summary of XRD profiles performed in order to study the compositional change on the substrate at each step. Those are as-purchased, air annealing, plasma treatment, reduction before growth, CNT growth and CNT oxidation after growth. As-purchased bare substrate, all peaks came from Inconel 600. Then, some oxide phases, such as NiCr_3_O_4_, Cr_2_O_3_ and NiO, were detected after thermal annealing at 725 °C for 10 min under air. The detected phases were not changed after the successive Ar plasma treatment for 30 min at 500 °C. The plasma power was 10.5 W.

Before the growth of CNT, the oxide phases disappeared because we changed the inlet gases of the CVD chamber to make reduction environment. From this result, we can expect the important factor to yield CNT would be a surface morphology in a nanoscale. After the growth of CNT for 30 min at 525 °C, we can clearly observe the diffraction peaks at 26° and 43° corresponding to (002) and (100) of the hexagonal graphite structure, respectively. In addition, we intentionally oxidized the CNT sample at 725 °C for 10 min to remove and investigate the surface state. The obtained XRD profile showedthe same pattern as that of annealed or plasma pretreated sample.

According to the investigation about the initial CNT growth stage, we can briefly conclude that the growth of CNT can be facilitated by the pretreatment consisting of thermal annealing and plasma ion bombardment due to the modified surface morphology having catalytic nanoparticles.

### 3.2. Growth Optimization of CNT on Inconel 600 Substrate

**Figure 5:**
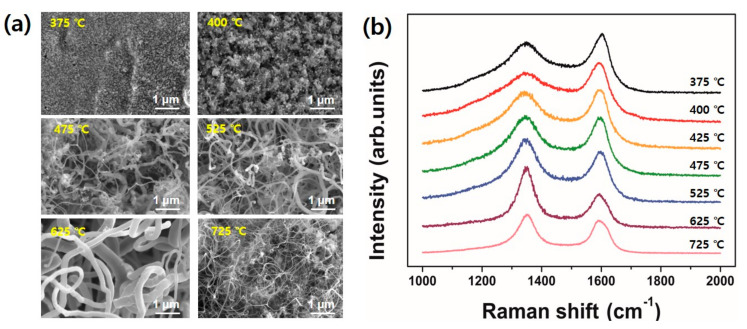
(**a**) SEM images showing the different morphologies of carbon nanotubes (CNTs) grown at specified temperature. The plasma power was kept at 10.5 W; (**b**) Raman spectra of the CNT samples in (a).

In this section, we demonstrate the results of growth optimization of CNT by varying the growth temperature and plasma power. First, we changed the growth temperature from 375 °C to 725 °C while plasma power was kept at 10.5 W. Figure 5a is SEM images showing the different morphologies of CNT grown at specified temperature and their corresponding Raman profiles are displayed in Figure 5b. At the growth temperature of 375 °C and 400 °C, it is hard to observe tubular structures in the SEM images although the samples produce a typical Raman spectra of multi-walled CNTs. As the growth temperature increases, for instance 475 °C and 525 °C, relatively long CNTs are observed. The value of I_D_/I_G_ ranges from 0.86 to 1.23. Although higher value of I_D_/I_G_ implies lower crystallinity of the nanotube structure, the value ranges obtained in this study is not critical range to reflect the structural degree of the CNTs as we confirmed from our previous works [5]. It is interesting that the CNT sample grown at 625 °C is a mixture of very thick and thin tubes. On the other hand, the CNTs grown at 725 °C looks thinner than other tubes obtained in this study. In addition, while the value of I_D_/I_G_ from CNTs grown at 625 °C reaches 1.85, but CNTs grown at 725 °C shows a relatively lower I_D_/I_G_ value of 1.15. At the present, it is expected the difference may be related to the morphology of catalytic nanoparticles on the substrate surface induced by the pretreatments. In addition, enhanced surface diffusion at higher growth temperature would produce smaller nanoparticles with the aid of plasma ion bombardment and finally nucleate the thinner CNTs. However, further investigation is required for extensive understanding.

**Figure 6:**
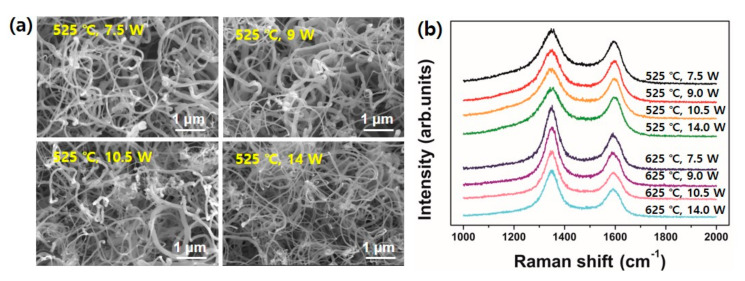
(**a**) SEM images showing the different morphologies of CNT grown at specified plasma power. The growth temperature was kept at 525 °C; (**b**) Raman spectra of the CNT samples in (a).

The growth temperature was fixed at 525 °C when we investigated the effect of plasma power on CNT growth. The results are shown in Figure 6a. As the plasma power get increased, the tube diameters appeared thinner. It can be clearly observed when we compare the CNT samples between 10.5 W and 14 W of applied plasma power. Here, it is expected the plasma ion bombardment with high power results in smaller catalytic nanoparticles as we confirmed in Figure 2c. It is plausible because a higher powered plasma generally possesses higher plasma density and ion bombardment energy as well. Figure 6b shows Raman spectra obtained from the samples grown at 525 °C and 625 °C with different plasma powers. It is interesting the value of I_D_/I_G_ at the same growth temperature shows almost the same value regardless of the applied plasma power. It implies, from a CNT structural point of view, the effect of plasma power applied at the pretreatment stage is relatively small compared to the growth temperature. However, it is noteworthy the plasma pretreatment is a crucial to form a catalytic nanoparticles and thus facilitate the nucleation of CNTs at an initial growth stage.

**Figure 7:**
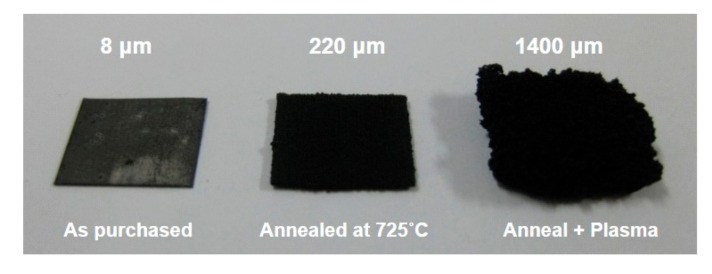
Digital image showing the effect of substrate pretreatment on CNT growth.

Finally, we can summarize the optimized pretreatment condition is a mixed process of thermal annealing at 725 °C for 10 min and successive plasma ion bombardment with Ar gas at 500 °C with 10.5 W for 30 min. Figure 7 clearly shows the effect of substrate pretreatment of Inconel 600 on CNT growth. Again, the growth was performed at 525 °C for 30 min.

**Figure 8:**
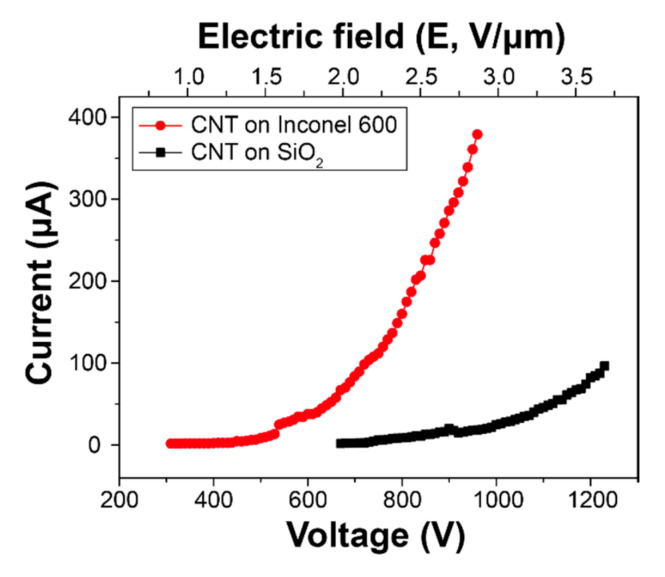
I–V characteristics of the CNTs grown on SiO_2_ and Inconel 600 substrates.

In order to demonstrate the advantage of direct growth of CNTs on metallic substrate, we performed a field emission (FE) measurement using the CNTs grown on conventional SiO_2_ wafer and Inconel 600. Briefly, the FE measurement was performed using a simple diode configuration and the distance between CNT top surface and the anode was kept at 500 μm. The chamber was evacuated below 5 × 10^−7^ Torr. Figure 8 shows current–voltage curves of the CNTs grown on SiO_2_ wafer and Inconel 600. Because FE phenomenon is based on electron tunneling, we estimated field enhancement factor (*β*) using the Fowler-Nordheim (FN) plot from Figure 8. The *β* can be calculated as following relation [17,18].

(1) $$\beta =-\;\frac{B\;\times \;\varphi^{\frac{3}{2}}\;\times D}{slope}$$

where, *B* is the FN constant (6.83 × 10^3^ V eV^−3/2^ μm^−1^), *φ* is a work function of CNTs (5 eV) [19], *D* is distance between CNTs and anode (500 μm) and slope of the F–N plot. In this study, while the *β* from CNTs grown on SiO_2_ wafer was estimated 4957, the *β* from CNTs on Inconel 600 shows 8177. The significant improvement in the FE performance would be a result of direct growth on metallic substrate.

According to the investigation about the initial CNT growth stage, we can briefly conclude that the growth of CNT can be facilitated by the pretreatment consisting of thermal annealing and plasma ion bombardment due to the modified surface morphology having catalytic nanoparticles.

### 3.3. Comparison of CNT Growth Behavior with Other Ni-Based Superalloys

Based on the results of growth behavior of CNT on Inconel 600, we compare the effect of pretreatments with other metallic substrates, Ni-based superalloys. Ni plate was also used for a comparison. As shown in Table 1, Invar 42 is a representative low thermal expansion alloy composed of mainly Ni and Fe. Hastelloy C276 is a Ni–Mo–Cr alloy with excellent corrosion resistance.

Because Ni is a common element in the alloys, it is possible to directly grow CNTs on the alloy surface. Thus, it is important to explore the change of surface state by changing the pretreatments in order to achieve efficient growth of CNTs as we investigated in Inconel 600.

**Figure 9:**
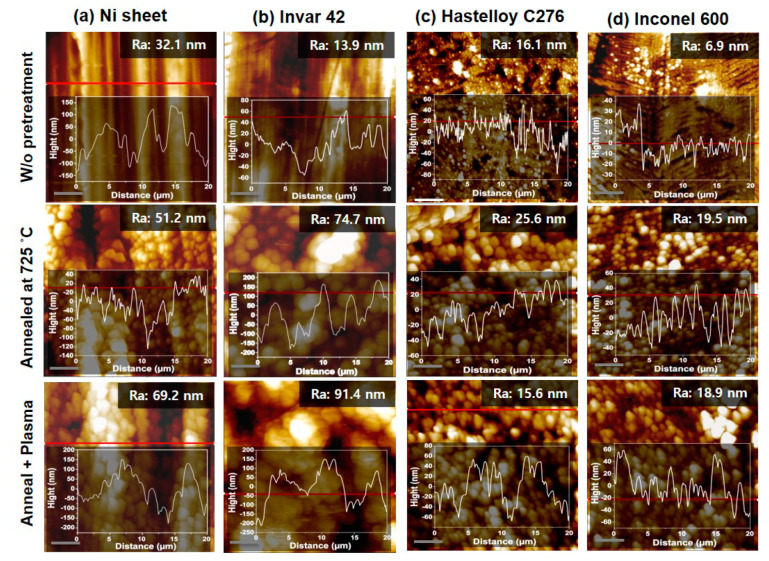
AFM results of (**a**) Ni sheet, (**b**) Invar 42, (**c**) Hastelloy C276 and (**d**) Inconel 600 with respect to different pretreatments. Each topographic image has height profile of the sample.

Figure 9 shows AFM topographic images of Ni sheet, Invar 42, Hastelloy C276 and Inconel 600 with respect to before pretreatment, thermal annealing at 725 °C and additional Ar plasma treatment with 10.5 W at 500 °C. It can be found from AFM images that the surface structure shows nanoparticle morphology and the quantitative surface roughness presented by arithmetical mean height (Ra) value slightly increased by the pretreatments. This well agrees with the height profiles inserted in each image. Using these substrates, we performed the CNT growth with different growth temperatures.

**Figure 10:**
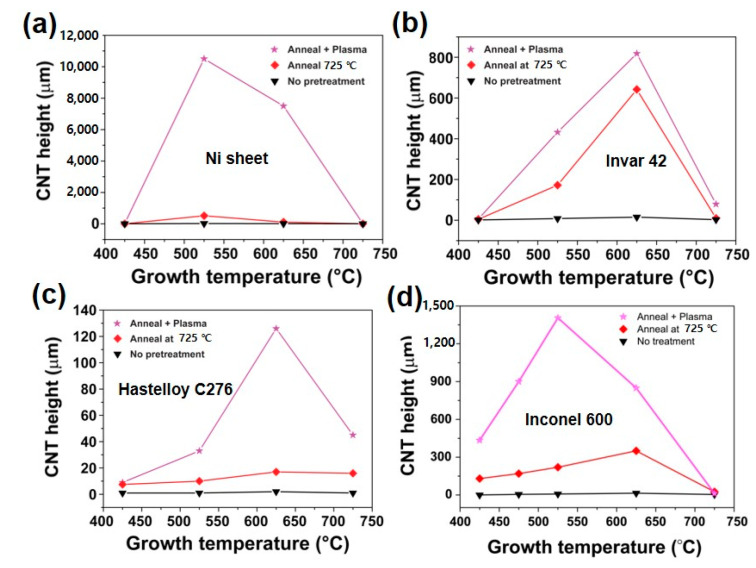
CNT growth results with respect to the growth temperature and pretreatment. (**a**) Ni sheet, (**b**) Invar 42, (**c**) Hastelloy C276 and (**d**) Inconel 600.

Figure 10 is a summary of CNT growth results. All substrates produced the highest CNT film thickness are undergone thermal annealing and additional Ar plasma treatment as we obtained in Inconel 600. The only difference is an optimal growth temperature, which produces the highest CNT forest thickness. In detail, the optimized growth temperature was determined at 525 °C for Ni and Inconel 600. On the other hand, the temperature is found to be 625 °C for Invar 42 and Hastelloy C276 substrates. The difference may be attributed to the existence of high temperature elements such as Mo and Cr in Invar 42 and Hastelloy C276. In particular, the poor growth of CNTs on Hastelloy C276 would be caused by a large content of Mo, which is inactive element in CNT growth.

**Figure 11:**
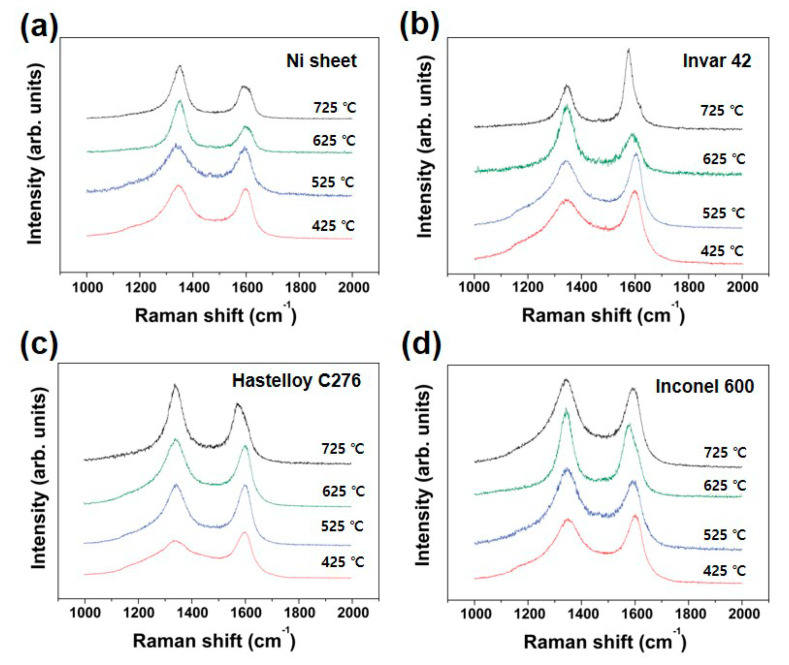
Raman spectra from CNTs grown with different growth temperature. (**a**) Ni sheet, (**b**) Invar 42, (**c**) Hastelloy C276 and (**d**) Inconel 600.

Figure 11 summarizes Raman spectra from CNTs grown using different substrates and growth temperature. All substrates were also treated with thermal annealing and successive Ar plasma treatment. Then, CNTs were grown at the optimized growth temperature. Although the CNTs grown at higher temperature show lower I_D_/I_G_ value, the obtained value is not sensitive to exactly reflect the structural difference of the CNTs. The situation is very similar to that of Inconel 600 as we confirmed in Figure 5 and Figure 6.

**Figure 12:**
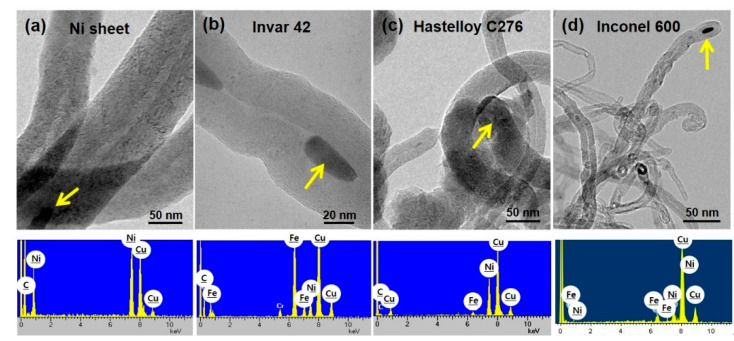
TEM images showing the detailed nanostructures of CNTs. (**a**) Ni sheet, (**b**) Invar 42, (**c**) Hastelloy C276 and (**d**) Inconel 600. EDS profiles are recorded from the catalytic nanoparticles indicated by arrows in each image.

In order to investigate the detailed nanostructures, we performed TEM observation using the representative CNT samples from each substrate and the results are shown in Figure 12. High resolution TEM images of the CNTs reveal the graphitic layers along the tube axis. The main elements of the catalysts designated by yellow arrows are confirmed as Ni and Fe as presented in EDS results. This well agrees with the chemical composition of the alloys. Cu peak is attributed to the TEM grid.

Finally, we believe that the pretreatments yields highly efficient growth of CNTs on Ni-based alloys by the formation of catalytic nanoparticles with appropriate roughened surface.

## 4. Discussion

In order to address the effect of substrate pretreatments on CNT growth, we investigated the early stage of CNTs growth on Inconel 600 and compared the growth results to other Ni-based superalloys such as Invar 42 and Hastelloy C276. As for the pretreatments, thermal annealing was performed at 725 °C for 10 min and Ar plasma pretreatment was subsequently undergone with 10.5 W at 500 °C for 30 min. The annealed and plasma-pretreated substrates exhibited roughened surface morphologies with nanoparticles over the surface and finally enhanced growth of CNT was observed.

The CNTs on Inconel 600 substrate showed superior field emission properties, such as lower turn-on voltage and higher field enhancement factor, over the CNTs grown on SiO_2_ substrate. This is clear evidence of enhanced electric conductivity at the interface between CNTs and the Inconel substrate. The optimized growth temperatures were determined at 525 °C for Ni and Inconel 600 and 625 °C for Invar 42 and Hastelloy C276 substrates, respectively. The difference of optimal growth temperature is attributed to the different chemical composition in the alloys. Finally, it can be explained that the formation of roughened oxide surface caused by the pretreatments would facilitate the nucleation and growth of the CNTs.

## Figures and Tables

**Table 1:** Chemical composition of Ni-based superalloys (wt.%).

Name	Ni	Cr	Mo	Mn	Fe	Etc.
Inconel 600	72	14–17	–	<1	6–10	Bal.
Invar 42	<41	–	–	<0.4	Bal.	56–58
Hastelloy C276	57	14.5–16.5	15–17	<1	4–7	Bal.

**Table 2:** Quantitative energy dispersive spectroscopy (EDS) results from 4 points in Figure 1 (wt.%).

	Ni	Cr	Fe	Etc.
Inconel 600	72	14–17	6–10	Bal.
Point 1	75.67	14.62	8.46	1.26 (O)
Point 2	81.29	2.73	12.95	3.04 (O)
Point 3	73.21	14.83	7.09	3.41 (O)
Point 4	83.49	2.06	14.46	3.13 (O)
